# Excessive hypercholesterolemia in pregnancy impairs the cardiovascular health of the adult female and male offspring

**DOI:** 10.1042/BSR20253861

**Published:** 2026-01-09

**Authors:** Murilo E. Graton, Amanda A. de Oliveira, Aryan Neupane, Raven Kirschenman, Anita Quon, Floor Spaans, Christy-Lynn M. Cooke, Sandra T. Davidge

**Affiliations:** 1 Department of Obstetrics and Gynecology, University of Alberta, Edmonton, AB, Canada; 2 Women and Children’s Health Research Institute, University of Alberta, Edmonton, AB, Canada; 3 Department of Physiology, University of Alberta, Edmonton, AB, Canada

**Keywords:** adult offspring, cardiac function, developmental origins of health and disease, pregnancy complications, sex differences, vascular function

## Abstract

Excessive hypercholesterolemia in pregnancy is a risk factor for the development of preeclampsia, but its ensuing impact on offspring cardiovascular health remains not fully understood. In the present study, Sprague Dawley rats were fed a control or high cholesterol diet (HCD) from gestational days 6–20 (term=22 days). Female and male offspring were aged to adulthood (4 months old), and *in vivo* and *ex vivo* cardiovascular function were assessed. In female offspring, body weight gain was greater in the HCD group compared with controls after 2 months of age. Blood pressure, echocardiographic parameters, cardiac capacity to recover from an ischemic insult, and endothelium-dependent vasodilation were similar between the female groups. However, vasoconstriction to a thromboxane analog (U46619) was reduced in thoracic aortas, but not in mesenteric, coronary, or carotid arteries, from the HCD females compared with controls, and was associated with lower phosphorylated myosin phosphatase target subunit 1 (MYPT1^Thr855^) levels. In male offspring, body weight gain, blood pressure, and echocardiographic parameters were similar between the groups, and there was no impact of HCD on vasoconstriction to U46619 or vasodilation in mesenteric arteries, carotid arteries, or thoracic aortas. However, endothelium-dependent vasodilation was reduced in coronary arteries of HCD males compared with controls and was associated with increased reactive oxygen species levels. There was also reduced cardiac capacity of the HCD males to recover from an ischemic insult. In summary, excessive hypercholesterolemia in pregnancy impaired the cardiovascular health of adult female and male offspring, but the mechanisms and vascular beds affected were specific to each sex.

## Introduction

Cardiovascular disease is the leading cause of death in the world [1]. Among the traditional cardiovascular disease risk factors, it is becoming increasingly recognized that being born from a complicated pregnancy can heighten the chance of having cardiovascular disease later in life [2,3], as theorized by the concept of the developmental origins of health and disease [4]. While hypercholesterolemia is a traditional cardiovascular disease risk factor [5], during pregnancy, the transient development of hypercholesterolemia is required to support the normal growth and development of the fetus [6]. However, excessively high circulating levels of cholesterol in pregnancy have been associated with an increased risk of developing preeclampsia [7], a common and potentially life-threatening complication of pregnancy. Preeclampsia affects ~3–8% of all pregnancies and is a major cause of maternal–fetal morbidity and mortality [8,9]. It can also negatively affect the health of the offspring throughout the lifespan [9]. In fact, adult offspring born from preeclamptic pregnancies are more susceptible to having an adverse cardio- or cerebrovascular event [10], being obese [11], or being diagnosed with neurological disorders [12]. In addition, in animal models, exposure to a preeclampsia-like phenotype during pregnancy resulted in various complications in the adult offspring, including adverse cardiovascular [13], metabolic [14], and neurodevelopmental [15] outcomes. However, the ensuing impact of excessive hypercholesterolemia in pregnancy on offspring health later in life has not been fully elucidated.

A high cholesterol diet (HCD) during pregnancy in rodent models leads to excessive *pregnancy-specific* hypercholesterolemia and induces a preeclampsia-like phenotype, including high blood pressure and proteinuria [16,17]. Moreover, our group recently demonstrated that an HCD in pregnancy increased the number of late resorptions and reduced placental efficiency (increased placental weight without changes in fetal weight) in both fetal sexes [17]. In addition, others have demonstrated that a HCD during pregnancy and lactation resulted in growth-restricted pups, stiffening of the cerebral arteries, increased thromboxane A_2_ (TxA_2_)-mediated venous vasoconstriction, inflammation, oxidative stress, and perinatal stroke in the juvenile offspring [18], while increasing heart rates in adult offspring [19], independent of offspring sex. However, the mechanisms by which excessive *pregnancy-specific* hypercholesterolemia could affect the vascular and cardiac health of the adult offspring remain to be assessed.

Endothelial dysfunction plays a key role in the development of cardiovascular disease and is characterized by an imbalance between vasoconstriction and vasodilation [20]. The vascular endothelium regulates vascular tone, in part, via the production and release of vasorelaxant substances, such as nitric oxide (NO), and vasoconstrictor substances, such as TxA_2_ [21]. NO, a potent vasodilator molecule important for regulation of blood pressure [22], can be inactivated by its reaction with superoxide, a common type of reactive oxygen species (ROS), leading to the formation of peroxynitrite, a potent oxidant and vasoconstrictor [23]. In contrast, TxA_2_ is an arachidonic acid metabolite involved in vasoconstriction via activation of thromboxane prostanoid (TP) receptors [24] and subsequent activation of downstream signaling pathways, including the activation of the Rho-kinase and protein kinase C-potentiated myosin phosphatase inhibitor of 17 kDa (PKC/CPI-17) pathways [25]. These downstream signaling pathways are shared with other vasoconstrictors, such as the α-1 adrenergic receptor agonist phenylephrine (PE) [26]. The Rho-kinase and PKC/CPI-17 pathways have been shown to contribute to impaired vascular function in adult offspring born from other models of pregnancy complications, such as prenatal hypoxia [27–29] or gestational diabetes [30]. However, the impact of excessive maternal hypercholesterolemia in pregnancy on vascular function in adult offspring remains to be assessed.

A compromised *in utero* environment can also negatively affect the cardiac health of the offspring. For example, in a rat model, prenatal hypoxia exposure leads to cardiac dysfunction in adult female and male offspring [31], with left ventricular diastolic dysfunction and pulmonary hypertension in both male and female offspring and left ventricular hypertrophy in male offspring only [32]. In addition, exposure to prenatal hypoxia reduced the cardiac capacity to recover from an ischemic insult in both male and female offspring and lowered the post-ischemic cardiac power in male offspring only [33]. However, it is not known whether excessive pregnancy-specific hypercholesterolemia also affects cardiac function in adult offspring.

Therefore, in the present study, we investigated if excessive hypercholesterolemia in pregnancy affects cardiac and vascular function in adult female and male offspring and assessed the potential mechanisms. We hypothesized that offspring born from an excessive hypercholesterolemic pregnancy will have impaired cardiovascular function in adulthood.

## Materials and methods

### Animals and experimental design

Female and male (19 females and 6 males for breeding) Sprague Dawley rats (3 months old) were acquired from Charles River Laboratories (Kingston, NY, U.S.A.). Rats were housed in a controlled environment, with a room temperature of 22±1°C and light–dark cycles of 14:10 h. The environment was enriched with crinkle paper, a wood-chewing block, and a plastic tube. Rats were housed in pairs, had *ad libitum* access to standard rat chow and water, and were acclimated in the housing room for at least one week before breeding. Next, female and male rats were mated overnight and checked for pregnancy in the following morning. The presence of sperm in a vaginal smear was considered gestational day (GD) 0 (term pregnancy=22 days). Once pregnancy was confirmed, dams were separated and single-housed during pregnancy. On GD 6, dams were arbitrarily assigned to a control diet (CD; standard rodent chow; PicoLab Rodent Diet 20 5053) or a HCD (Modified LabDiet^®^ 5001 with 2% cholesterol and 0.5% sodium cholate) until GD 20. This rat model of excessive hypercholesterolemia in pregnancy has been previously characterized by our group and others [16–19,34], and displays a preeclampsia-like phenotype, including the classical signs of preeclampsia (i.e. high blood pressure and proteinuria), as well as uteroplacental dysfunction [17]. From GD 21 and during lactation, both CD and HCD dams received CD. To standardize postnatal conditions, litter size was reduced at birth (GD 22) to eight pups (four males and four females from the same litter). On postnatal day 21, the offspring were weaned, separated by sex, double-housed under standard conditions, and received standard rodent chow until adulthood (4 months old). Body weights were collected from female and male offspring and averaged for each dam on postnatal days 1 and 21, as well as at 1, 2, 3, and 4 months of age. Body weight gain was calculated relative to body weight on postnatal day 1. At 4 months old, one female and one male offspring from each dam were selected arbitrarily and used to assess blood pressure with a non-invasive tail-cuff plethysmography system (see below). Next, animals were killed by exsanguination via cardiac puncture under isoflurane anesthesia (4% in 100% O_2_). Thereafter, the mesenteric arcade, the left carotid artery, the thoracic aorta, and the heart (to isolate the left anterior descending coronary artery) were immediately excised and placed into ice-cold HEPES-buffered physiological salt solution (PSS, in mmol/L: 10 HEPES, 142 NaCl, 4.7 KCl, 5.5 C_6_H_12_O_6_, 1.56 CaCl_2_, 1.17 MgSO_4_, 1.18 KH_2_PO_4_; pH 7.4). Arteries were dissected and cleaned of surrounding tissues for the assessment of *ex vivo* vascular function using wire myography (see below). In addition, arterial segments were frozen in optimal cutting temperature compound (Tissue Tek^®^; Sakura Finetek, Torrance, CA, U.S.A.) for cryosectioning or snap-frozen for Western blotting. In another set of experiments, one female and one male offspring from each dam were selected arbitrarily and used for the assessment of cardiac function by transthoracic echocardiography and isolated *ex vivo* heart function (see below).

### Blood pressure measurements

At 4 months of age, one female and one male offspring from each dam were trained daily in 30-min training sessions for three consecutive days before blood pressure measurements. Mean arterial blood pressure was assessed using a tail-cuff plethysmography system (CODA^®^; Kent Scientific Corporation, Torrington, CT, U.S.A.), and an average of at least ten blood pressure cycles was calculated [35].

### *Ex vivo* assessment of vascular function using wire myography

Vascular function was assessed in mesenteric arteries, carotid arteries, thoracic aortas, and coronary arteries using a wire myograph system (620M DMT, Copenhagen, Denmark), and data were recorded using LabChart software (version 8.1.13; AD Instruments, Colorado Springs, CO, U.S.A.).

Mesenteric (second-order), carotid (left common carotid artery), and coronary (left anterior descending artery) arteries (~2 mm rings) were mounted, in accordance with previously established protocols [28,36–38], on two 40-μm wires. Vessels were normalized to their optimal resting tension using a stepwise procedure (0.8× IC100; 13.3 kPa). After a 20-min equilibration period in PSS (pH 7.4, 37°C), vessels were stimulated with either PE (mesenteric arteries: 10 μmol/L; carotid arteries: 1 μmol/L) or 9,11-dideoxy-9α,11α-methanoepoxy prostaglandin F2α (U46619, a TxA_2_ analog; coronary arteries: 1 μmol/L) for 5 min. Next, vessels were washed thrice and allowed to rest for 10 min. Vessels were then re-stimulated with either PE or U46619 for 5 min and had their endothelium integrity tested by adding 3 μmol/L of methacholine (MCh) for 2 min. After a 30-min resting period, a cumulative concentration–response curve (CCRC) to U46619 (0.1 nmol/L–10 µmol/L; added in 2-min intervals, or until plateau) or PE (0.1 nmol/L –100 μmol/L; added in 2-min intervals, or until plateau) was performed. In the coronary arteries, only a CCRC to U46619 was performed, as this vascular bed presents a weak vasoconstrictor response to PE [39]. Vessels were then washed and allowed to rest for another 30 min. Thereafter, a CCRC to MCh (0.1 nmol/L–100 μmol/L; added in 2-min intervals) was performed in vessels pre-constricted with PE (10 µmol/L for mesenteric arteries or 1 µmol/L for carotid arteries) or U46619 (pre-calculated EC_80_ – effective concentration that produces 80% of the maximal response – for coronary arteries). Afterward, vessels were washed until resting tension, allowed to recover for at least 15 min, and stimulated with high K^+^ PSS (KPSS; 123 mmol/L) to assess maximum vascular smooth muscle vasoconstriction capacity (5 min for mesenteric arteries and 30 min for carotid and coronary arteries).

Thoracic aortas (~2 mm rings) were mounted, using a previously established protocol [38], on two stainless steel pins, and normalized by stretching to a resting tension of 30 mN (equivalent to a transmural pressure of 13.3 kPa). The PSS (pH 7.4, 37°C) was replaced every 15 min during the 1-h stabilization period. Vessels were then exposed to KPSS for 30 min, washed until resting tension, and allowed to recover for 10 min. Next, vessels were stimulated once with PE (1 μmol/L) for 10 min and exposed to MCh (3 μmol/L) for 2 min. Vessels were then washed and allowed to rest for 30 min before performing a CCRC to U46619 (0.1 nmol/L–10 µmol/L) or PE (0.1 nmol/L–100 μmol/L); both drugs were added in 2-min intervals or until plateau. After washing the vessels and a 30-min recovery time, a CCRC was performed to MCh (0.1 nmol/L–100 μmol/L; added in 2-min intervals) in vessels pre-constricted with PE (1 µmol/L).

All wire myography data were normalized to vessel length. Vasoconstriction was calculated from the vasoconstrictor CCRCs (in mN/mm), while vasorelaxation CCRCs were used to calculate percent vasorelaxation from the maximum vasoconstriction elicited during pre-constriction. Vasorelaxation and vasoconstrictor CCRCs were also used to summarize both the potency and efficacy of the agonists [i.e. area under the curve (AUC), in arbitrary units], as shown in the summary graphs. Vasoconstriction to KPSS was summarized as maximum vasoconstriction (in mN/mm).

### Western blotting in thoracic aortas

The main components of the Rho-kinase pathway, which is initiated by the activation of the TP receptor after its binding with U46619, including Rho-associated coiled-coil kinase 1 (ROCK1), Rho-associated coiled-coil kinase 2 (ROCK2), myosin phosphatase target subunit 1 phosphorylated at threonine 855 (pMYPT1), and myosin phosphatase target subunit 1 (MYPT1), were assessed by Western blotting. For this purpose, thoracic aortas were homogenized in lysis buffer (in mmol/L: 20 Tris, pH 7.4; 5 ethylenediaminetetraacetic acid; 10 tetrasodium pyrophosphate; 100 sodium fluoride; 2 sodium orthovanadate; 1% Nonidet P-40 (NP-40); 20 µg/mL phenylmethanesulfonyl fluoride; 1× protease inhibitor [87786, Halt™ Protease Inhibitor Cocktail (100×); ThermoFisher Scientific]). Total protein concentrations were determined using a Pierce™ BCA Protein Assay kit (23227; ThermoFisher Scientific). Samples (50 µg for ROCK1 and ROCK2 or 100 µg for TP receptor, pMYPT1, and MYPT1) were loaded onto 6% (ROCK1, ROCK2, pMYPT1, and MYPT1) or 10% (TP receptor) sodium dodecyl sulfate–polyacrylamide gels, separated by electrophoresis (110 V, 1.5–2 h, 4°C), and transferred (100 V, 1 h, 4°C) onto 0.2 µm nitrocellulose membranes (Bio-Rad, Mississauga, ON, Canada). Total protein content was determined using LI-COR Revert™ 700 Total Protein Stain (926–11021; LI-COR Biosciences, Lincoln, NE, U.S.A.), in accordance with the manufacturer’s protocol. Next, membranes were washed [6.7% acetic acid and 30% methanol in phosphate-buffered saline (PBS), two times for 30 s] and destained (using 0.1 mol/L sodium hydroxide and 30% methanol in PBS, for 10 min). All membranes were blocked with a blocking solution (Rockland, Limerick, PA, U.S.A.) for 1.5 h at room temperature. Membranes were incubated overnight at 4°C with primary antibodies for the TP receptor (1:1,000, rabbit polyclonal; Cayman Chemical, Ann Arbor, MI, U.S.A.), ROCK1 (1:5,000, rabbit polyclonal, 21850–1-AP; Proteintech, Rosemont, IL, U.S.A.), ROCK2 (1:4,000, rabbit polyclonal, 21645–1-AP; Proteintech), pMYPT1^Thr855^ (1:250, rabbit polyclonal, 4563; New England Biolabs, Whitby, ON, Canada), or MYPT1 (1:3,000, mouse monoclonal, 66506–1-lg; Proteintech). All primary antibodies were diluted in 1× PBS with 0.1% Tween 20 (PBST). The following day, membranes were incubated with their corresponding secondary antibodies as follows: TP receptor, ROCK1, ROCK2, and pMYPT1: IRDye^®^ 800CW donkey anti-rabbit IgG; and MYPT1: IRDye^®^ 680RD donkey anti-mouse IgG – all used at a concentration of 1:10,000 and diluted in 25% blocking solution in 0.1% PBST (LI-COR Biosciences). Blots were visualized with a LI-COR Odyssey CLx Imaging System (LI-COR Biosciences), and band densitometry was quantified using ImageStudio software (version 3.1; LI-COR Biosciences). Reported values for TP, ROCK1, ROCK2, and MYPT1 were normalized to total protein staining, while reported values for pMYPT1^Thr855^ were normalized to MYPT1 levels. Of note, the TP receptor can be glycosylated to facilitate its trafficking to the cellular membrane, which results in the appearance of multiple bands [40]; thus, all bands were combined and quantified together. Membranes are available in the Supplementary Material (Supplementary Figures S3-S7).

### Immunofluorescence for endothelial NO synthase, inducible NO synthase, and 3-nitrotyrosine expression in coronary arteries

Coronary artery cryosections (9 µm) were thawed, fixed in cold acetone (−20°C) for 10 min, and washed thrice with PBS (pH 7.4) for 5 min each time. For quenching autofluorescence, slides were treated with sodium borohydride (1 mg/mL, diluted in PBS) for 20 min at room temperature and then washed thrice with PBS for 10 min each time. Slides were blocked with 5% donkey serum [diluted in 2% bovine serum albumin (BSA) in PBS with 0.1% Triton X-100] for 1.5 h at room temperature and washed thrice with PBS for 5 min each time. The sections were then incubated in a humidified chamber (overnight at 4°C) with mouse anti-inducible NO synthase (iNOS; 1:5, BD610432; BD BioSciences, Mississauga, ON, Canada), rabbit anti-endothelial NO synthase (eNOS; 1:50, sc-654; Santa Cruz Biotechnology, Dallas, TX, U.S.A.), or mouse anti-nitrotyrosine (1:50, NB11096877; Bio-Techne Canada, Toronto, ON, Canada) antibodies, all diluted in 2% BSA in PBS. For each slide, one section from a CD offspring was incubated with 2% BSA in PBS in the absence of the primary antibody, serving as the negative control. The next day, all sections were washed thrice with PBS for 5 min and incubated with the following secondary antibodies: AlexaFluor™ 488 donkey anti-mouse IgG (H+L) (for iNOS and 3-nitrotyrosine, A21202; ThermoFisher Scientific, Waltham, MA, U.S.A.) or AlexaFluor™ 546 donkey anti-rabbit IgG (H+L) (for eNOS, A11040; ThermoFisher Scientific), for 1 h at room temperature in a humidified chamber. All secondary antibodies were diluted (1:250) in 2% BSA in PBS supplemented with 4 nmol/L of 4',6-diamidino-2-phenylindole (D3571; ThermoFisher Scientific). Next, slides were washed thrice for 5 min with PBS, coverslipped with Vectashield^®^ Antifade Mounting Medium (H-1200 solution; Vector Laboratories Inc., Newark, CA, U.S.A.), sealed with clear nail polish, and stored protected from light until visualization.

### ROS detection in coronary arteries

Coronary artery ROS levels were assessed using dihydroethidium (DHE) fluorescent staining, a reproducible technique commonly used to determine intracellular ROS production [41] that has been previously used in various arteries from different animal models [17,28]. Briefly, coronary artery cryosections (9 µm) were washed thrice with Hank’s Balanced Salt Solution (HBSS; ThermoFisher Scientific) for 2 min each time. In a humidified chamber at 37°C, fresh HBSS was added for 10 min before incubation with 200 μmol/L DHE fluorescent probe (10057; Biotium, Fremont, CA, U.S.A.) for 30 min. For each slide, one section from a CD offspring was incubated with HBSS in the absence of DHE, serving as the negative control. Slides were then washed thrice with HBSS for 2 min each time, coverslipped, and imaged immediately.

### Analysis of immunofluorescent and stained images

All images from coronary artery sections were visualized using a 10× objective lens and captured using an Olympus IX81 microscope (Olympus Canada Inc., Toronto, ON, Canada) operated by cellSens Dimensions 1.9 software (Olympus Canada Inc.) with a CoolSNAP HQ2 CCD camera (Photometrics, Tucson, AZ, U.S.A.), as previously described [17]. One image per offspring and one offspring per sex per dam were used for the analysis. The mean fluorescence intensity (arbitrary units) of each image was analyzed using ImageJ software (1.53 [42]). Autofluorescence from the negative control was subtracted from all sections on the same slide, and the resulting values were reported. Representative images were adjusted for brightness and contrast using the same settings across all images.

### Assessment of cardiac function by transthoracic echocardiography

Female and male offspring were anesthetized using inhaled isoflurane (induction: 3%; maintenance: 1.8–2.5%; in 1.8 L/min O_2_), kept in the supine position on a controlled heating pad, and had the fur over the ventral thorax removed by shaving and then by application of a depilatory cream. Then, *in vivo* cardiac function was assessed by transthoracic echocardiography using an ultrasound biomicroscope (Vevo^®^ 2100; VisualSonics, Toronto, ON, Canada) connected to a 13–24 MHz MS250 transducer, as previously described by our group [43]. Data were analyzed using Vevo Lab software (v5.6.1; VisualSonics), in which at least three consecutive cardiac cycles that were not affected by breathing motion were traced and averaged for each offspring.

### Assessment of cardiac function by exposure to *ex vivo* ischemia/reperfusion insult

Cardiac function was assessed as the capacity to recover from an *ex vivo* ischemia/reperfusion (I/R) insult (modeling a secondary hit), using an isolated working heart preparation with a Hugo Sachs Elektronik data acquisition system and Isoheart Software for Windows 10 (Harward Apparatus, March, Germany). As previously described [33], after animals were killed, hearts were excised and placed in ice-cold modified Krebs–Henseleit solution (in mmol/L: 120 NaCl, 4.7 KCl, 5 C_6_H_12_O_6_, 1.25 CaCl_2_, 1.2 MgSO_4_, 25 NaHCO_3_, 1.2 KH_2_PO_4_; pH 7.4). Hearts were mounted on an aortic cannula, ligated with silk sutures, and perfused in retrograde Langendorff mode with modified Krebs–Henseleit solution (gassed with 95% O_2_ and 5% CO_2_; 37°C; pH 7.4), as the left atrium was cannulated and ligated with silk sutures. Next, the retrograde flow was closed, the anterograde flow was opened, and palmitate fatty acid (1.2 mmol/L) was added to the modified Krebs–Henseleit solution. The pressure at the cannulated left atrium was equivalent to 11.5 mmHg; the buffer then passed to the left ventricle, from which it was spontaneously ejected through the aortic cannula against a pressure equivalent to 80 mmHg (afterload). Measurements of cardiac function were recorded every 10 min for a total period of 90 min, which included 30 min of aerobic perfusion, 20 min of global ischemia (no-flow ischemia), and 40 min of aerobic reperfusion. Cardiac function was expressed as a percentage of recovery from baseline.

### Statistical analysis

All data are reported as means±standard error of the mean. Graphs were generated using GraphPad Prism, version 10.3.1 for macOS (GraphPad Software, Boston, MA, U.S.A.). Outliers were identified using Grubb’s test and removed from the final statistical analyses. For the wire myography data, the CCRCs were summarized using the AUC. All data were analyzed using an unpaired Student’s t-test; *n* represents the number of offspring per group (one male and one female offspring/dam/group), and a *P*<0.05 was considered significant.

## Results

### HCD in pregnancy was associated with greater body weight gain *only* in the female offspring

In the female offspring, body weights were similar between groups on postnatal day 1 (CD: 6.5±0.1; HCD: 6.6±0.2; *n*=8–10/group). However, while body weight gain was not different between groups on postnatal day 21 and at 1 month of age, after 2 months of age, body weight gain was significantly greater in the HCD female group compared with CD females (Table 1). Conversely, in the male offspring, body weights were similar between groups on postnatal day 1 (CD: 6.6±0.1; HCD: 6.9±0.2; *n*=8–10/group) as was the body weight gain until adulthood (Table 1).

**Table 1:** Body weight gain (g) and mean arterial pressure (mmHg) of the female and male adult offspring born from CD or HCD pregnancies

	CD	HCD	Unpaired t-test
**Female offspring**			
**Body weight gain**			
Postnatal day 21	51.5±1.2 (*n*=10)	54.4±0.9 (*n*=8)	*P*=0.1020
1 month old	93.0±1.9 (*n*=10)	101.1±3.6 (*n*=8)	*P*=0.0505
2 months old	230.8±3.6 (*n*=10)	255.0±7.0 (*n*=8)	***P*=0.0051**
3 months old	270.7±4.1 (*n*=10)	290.2±7.7 (*n*=8)	***P*=0.0320**
4 months old	288.3±6.4 (*n*=10)	317.7±10.2 (*n*=8)	***P*=0.0224**
**Blood pressure (at 4 months of age)**			
Mean arterial pressure	98.5±3.3 (*n*=11)	101.9±2.5 (*n*=8)	*P*=0.4604
**Male offspring**			
**Body weight gain**			
Postnatal day 21	55.2±1.6 (*n*=10)	57.3±0.8 (*n*=8)	*P*=0.3302
1 month old	104.9±2.1 (*n*=10)	109.7±1.8 (*n*=8)	*P*=0.1135
2 months old	404.4±7.3 (*n*=10)	414.1±7.0 (*n*=8)	*P*=0.3627
3 months old	521.7±9.6 (*n*=10)	536.5±8.5 (*n*=8)	*P*=0. 2806
4 months old	583.3±13.7 (*n*=10)	588.9±13.0 (*n*=8)	*P*=0.7767
**Blood pressure (at 4 months of age)**			
Mean arterial pressure	111.7±4.8 (*n*=11)	108.3±3.7 (*n*=8)	*P*=0.6055

Data are presented as means±SEM and were analyzed with an unpaired Student’s t-test (*n*=8–11/group; one offspring/sex/dam/group).

Bold values represent *P*<0.05.

SEM, standard error of the mean.

At 4 months of age, the mean arterial blood pressure was not different between the CD and HCD groups, regardless of offspring sex (Table 1).

### Vasoconstriction to U46619 was reduced in aortas of *only* the adult female HCD offspring

**Figure 1:**
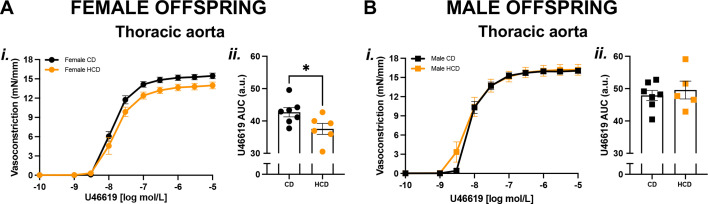
U46619-mediated vasoconstriction was reduced in thoracic aortas from *only* the female adult HCD offspring. CCRCs to the TxA_2_ analog U46619 (**i**) in thoracic aortas from adult female (A; circles) and male (B; squares) offspring born from CD (black symbols) pregnancies or HCD (orange symbols) pregnancies. Data were summarized as AUC (ii), presented as means±SEM, and analyzed with an unpaired Student’s t-test, *n*=5–8/group (one offspring/sex/dam/group). *P<0.05. Abbreviations: a.u., arbitrary units; SEM, standard error of the mean.

Vasoconstriction to U46619 was reduced in aortas from adult female (Figure 1A), but not male (Figure 1B), HCD offspring compared with CD controls, with no differences observed in vasoconstriction to U46619 in mesenteric, carotid, or coronary arteries (Supplementary Table S2). In addition, KPSS- and PE-mediated vasoconstriction responses were not different between CD and HCD female offspring in all studied vascular beds (Supplementary Tables S1 and S3). In adult male CD and HCD offspring, vasoconstriction responses to KPSS (Supplementary Table S1), U46619 (Supplementary Table S2), and PE (Supplementary Table S3) were similar between groups in all vascular beds.

**Figure 2:**
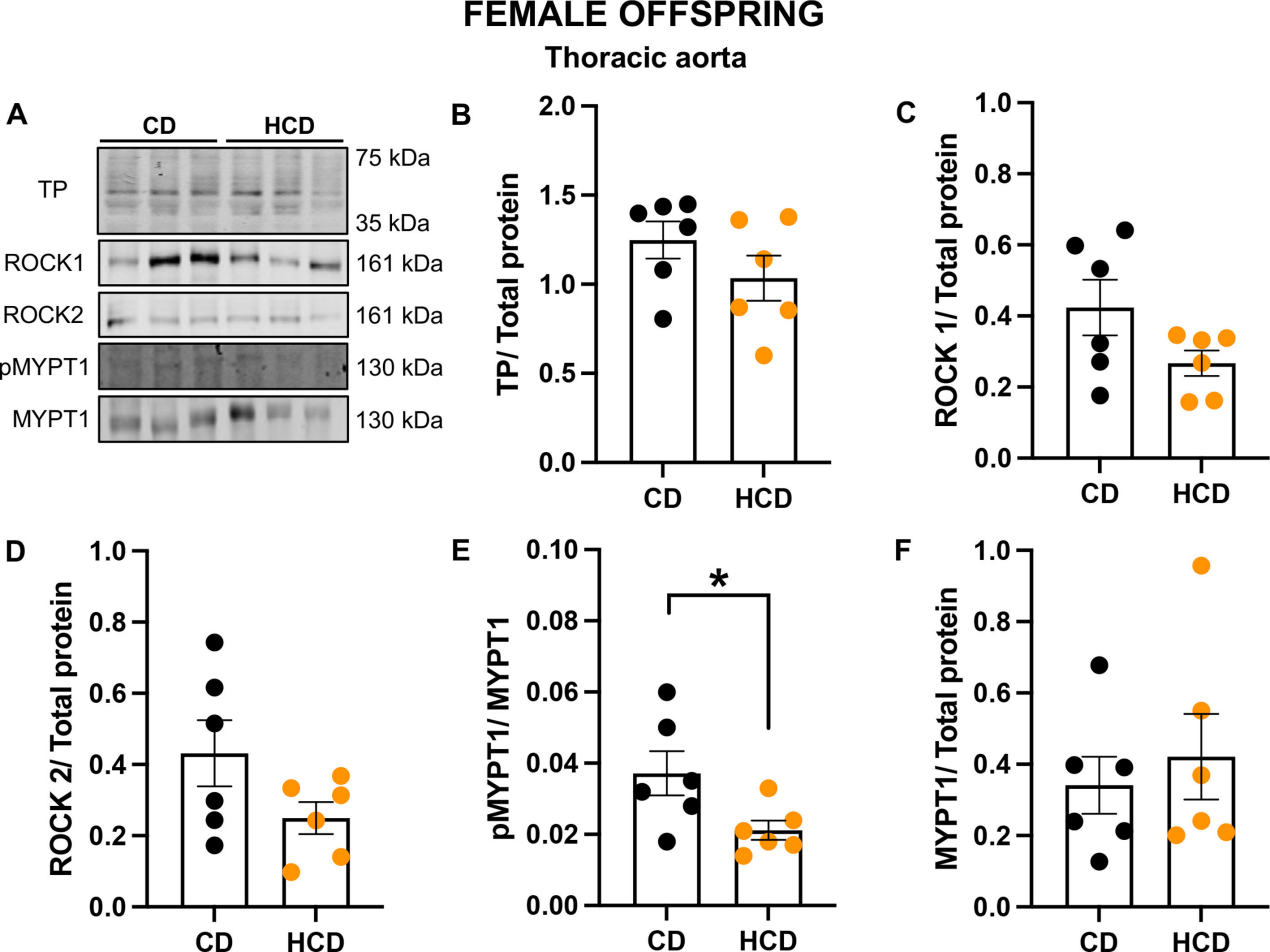
HCD in pregnancy reduced the phosphorylation of MYPT1 in thoracic aortas from the adult female offspring. Representative blots (**A**) and densitometry of TP receptor (B; normalized to total protein), ROCK1 (C; normalized to total protein), ROCK2 (D; normalized to total protein), pMYPT1 (E; normalized to MYPT1), and MYPT1 (F; normalized to total protein) in thoracic aortas from the adult female offspring born from CD (black symbols) or HCD (orange symbols) pregnancies. Data are presented as means±SEM and were analyzed with an unpaired Student’s t-test (*n*=6/group; one offspring/dam/group). *P<0.05. Abbreviation: SEM, standard error of the mean.

As only aortas from female HCD offspring exhibited impaired vasoconstriction to U46619, subsequent molecular analysis was conducted exclusively in this vascular bed to assess potential changes in the Rho-kinase pathway. The relative protein expression levels of pMYPT1^Thr855^ over total MYPT1 expression were reduced in HCD female offspring compared with CD controls (Figure 2A,E) and were unchanged in male offspring (Supplementary Figure 1). No changes were observed in the protein expression levels of the TP receptor (Figure 2A,B), ROCK1 (Figure 2A,C), ROCK2 (Figure 2A,D), or MYPT1 (Figure 2A,F).

### Vasodilation to MCh was reduced in coronary arteries of *only* the adult male HCD offspring

**Figure 3:**
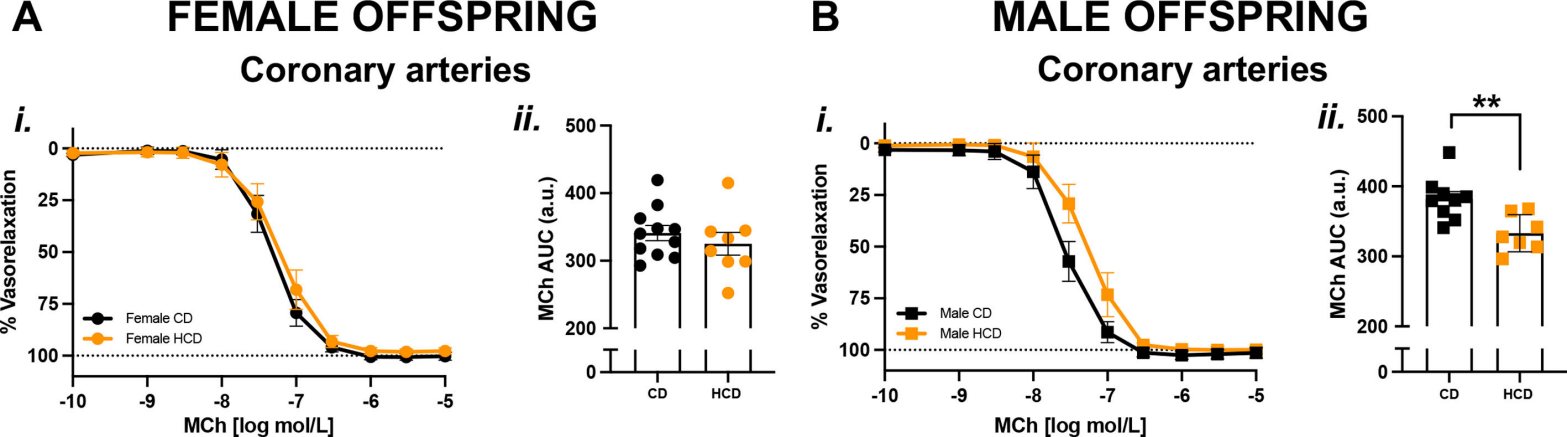
Endothelium-dependent vasorelaxation was impaired in coronary arteries from *only* the male HCD offspring. CCRCs to MCh (**i**) in coronary arteries from the adult female (A; circles) and male (B; squares) offspring born from CD (black symbols) or HCD (orange symbols) pregnancies. Data are summarized as AUC (ii), presented as means±SEM, and analyzed with an unpaired Student’s t-test (*n*=7–10/group; one offspring/sex/dam/group). **P<0.01. Abbreviations: a.u., arbitrary units; SEM, standard error of the mean.

Vasodilation responses to MCh were reduced in the coronary arteries of the male (Figure 3B), but not female (Figure 3A), HCD offspring compared with CD controls. In contrast, in mesenteric arteries, carotid arteries, and aortas, vasodilation responses to MCh were similar between the CD and HCD male and female offspring groups (Supplementary Table S4).

**Figure 4:**
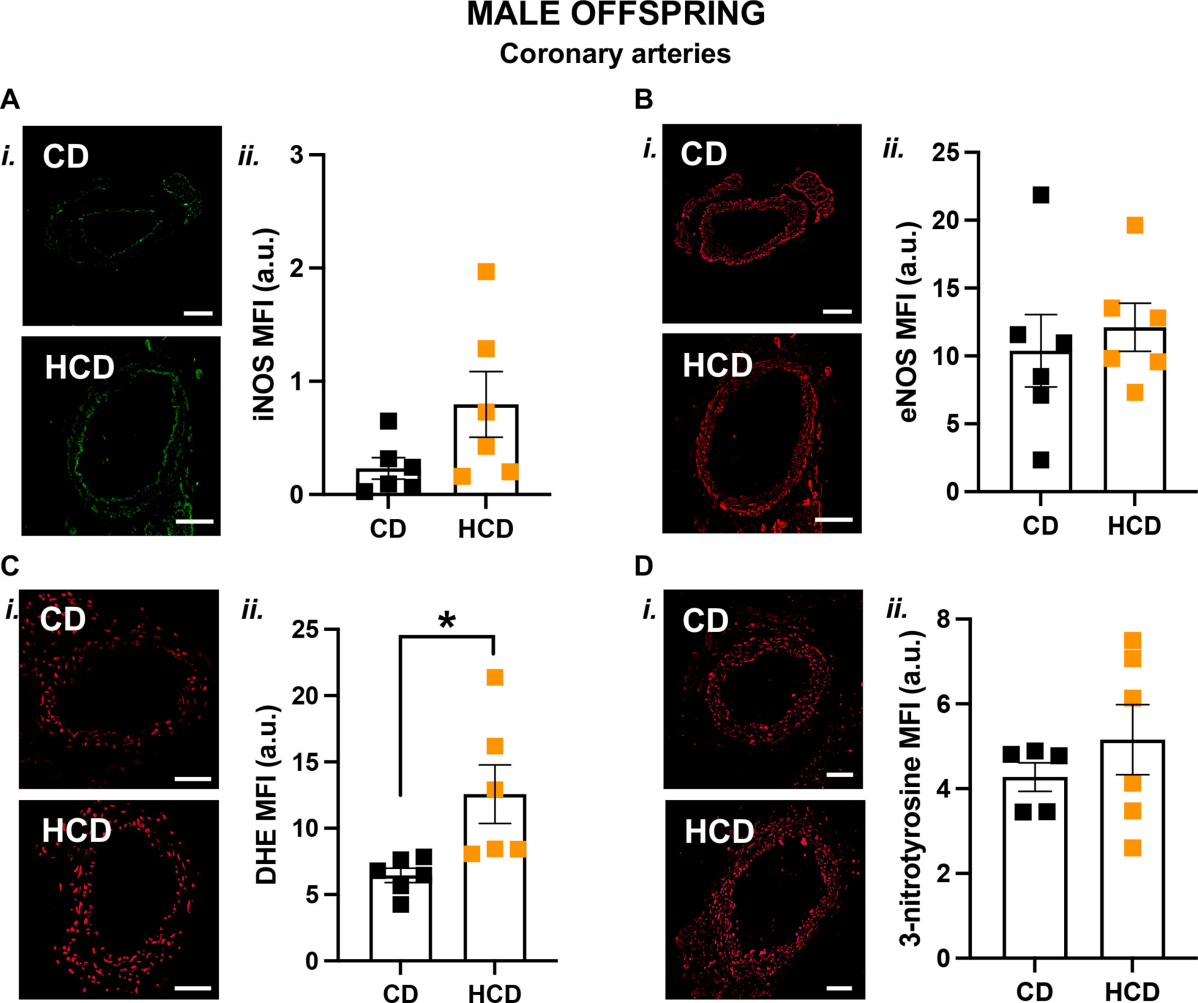
A HCD in pregnancy increased ROS levels in coronary arteries from the adult male offspring. Representative images (**i**) and quantitative analysis (ii) of iNOS (**A**), eNOS (**B**), DHE (**C**), and 3-nitrotyrosine (**D**) in coronary artery sections from adult male offspring born from CD (black symbols) or HCD (orange symbols) pregnancies. Data are presented as means±SEM and were analyzed with an unpaired Student’s t-test (*n*=5–6/group; one offspring/dam/group). *P<0.05. Abbreviations: a.u., arbitrary units; MFI, mean fluorescent intensity; SEM, standard error of the mean.

Since only the coronary arteries of male HCD offspring showed impaired vasodilation to MCh, subsequent molecular analysis was conducted exclusively in this vascular bed. ROS levels were increased in HCD male offspring compared with CD males (Figure 4C), with no differences observed in the levels of iNOS (Figure 4A), eNOS (Figure 4B), or 3-nitrotyrosine (Figure 4D) between groups. ROS levels were similar in the coronary arteries of adult female offspring (Supplementary Figure 2).

### Cardiac tolerance to an I/R insult was reduced in *only* the adult male HCD offspring

**Figure 5:**
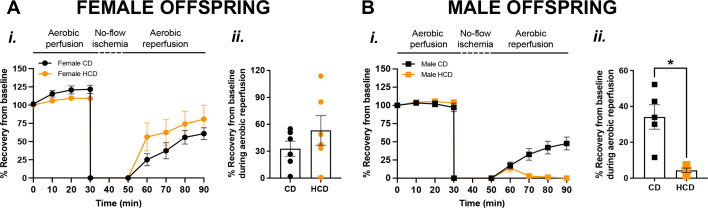
Adult male offspring born from HCD pregnancies have impaired cardiac recovery after I/R insult. Percentage of recovery from baseline during 30 min of aerobic perfusion, followed by 20 min of no-flow ischemia and 40 min of aerobic reperfusion (**i**), and percentage of cardiac recovery from baseline during aerobic reperfusion (ii) in isolated hearts from adult female (A; circles) and male (B; squares) offspring born from CD (black symbols) or HCD (orange symbols) pregnancies. Data are presented as means±SEM and were analyzed with an unpaired Student’s t-test (*n*=4–6/group; one offspring/sex/dam/group). *P<0.05. Abbreviation: SEM, standard error of the mean.

*In vivo* cardiac left ventricular morphology and function were similar between the CD and HCD groups, regardless of offspring sex (Supplementary Tables S5 and S6). However, after an *ex vivo* I/R insult (modeling a secondary hit), the percentage of cardiac recovery from baseline was lower in male HCD offspring compared with CD offspring (Figure 5B), with no differences observed in female groups (Figure 5A).

## Discussion

In the present study, we assessed the long-term impact of exposure to a pregnancy complicated by excessive hypercholesterolemia on cardiovascular function in the adult female and male offspring. In female offspring, we demonstrated that vasoconstriction responses to U46619 were reduced in aortas of HCD females compared with controls, which could be attributed to a reduction in the phosphorylation of MYPT1. In contrast, in male offspring, vasodilation responses to MCh were lower in the coronary arteries of HCD males compared with controls. And while *in vivo* cardiac function (assessed by echocardiography) was similar between adult male and female CD and HCD offspring, the capacity to recover from an I/R insult (modeling a secondary hit) was reduced in *only* the adult male, but not female, HCD offspring compared with controls. Altogether, these data support our hypothesis that being born from a pregnancy complicated by excessively high cholesterol levels affects cardiovascular function. Moreover, while exposed to the same suboptimal *in utero* environment, the vascular beds affected and the molecular mechanisms were specific to each sex.

We have observed that after 2 months of age, body weight gain was greater in female HCD offspring compared with controls, a difference that was not observed in male HCD offspring. In our animal model of excessive hypercholesterolemia in pregnancy leading to a preeclampsia-like phenotype, male and female fetal body weights were similar in the CD and HCD groups at the end of pregnancy [17]. This partially aligns with a population-based human study that showed an association between maternal hypercholesterolemia in pregnancy and increased body weight in the offspring up to 8 years of age, but not at the time of birth, in both females and males [44]. There are various potential mechanisms to account for sex differences in body weight gain, including energy metabolism, gut microbiota, fat distribution, and genetic predisposition [45] that were not the purpose of the current study but that may be of interest for follow-up studies, along with measurements of food intake and body composition. We also found that mean arterial blood pressures were similar between the CD and HCD groups, regardless of the offspring sex. As the mesenteric arteries contribute to the control of peripheral vascular resistance [46], and therefore the control and regulation of blood pressure, this is in line with the lack of impact of HCD in pregnancy on small resistance artery (i.e. mesenteric arteries) function in the adult offspring.

In only the female HCD offspring, vasoconstriction responses to U46619 were reduced in the thoracic aortas compared with controls, without impact on the mesenteric, carotid, or coronary arteries. Although a reduction in vasoconstriction may be interpreted as protective, vasoconstriction in the HCD group was lower than in the control group, suggesting an impairment of normal vascular function. Reduced vasoconstriction has also been observed in conditions, such as aging [47] and atherosclerosis [48]. The TxA_2_-mediated vasoconstriction is initiated by the activation of TP receptors, a G protein-coupled receptor [49]. TP receptors can be coupled to different G proteins, thus leading to the activation of distinctive intracellular pathways [50]. When activated, TP receptors coupled with G_q-11_ protein share with the α-1 adrenergic receptors (ligand for adrenergic agonists, such as PE) the same pathway involving the activation of the PKC/CPI-17 pathway, leading to vasoconstriction [25]. However, the majority of TP receptors are coupled to G_12-13_ protein, activating the Rho-kinase pathway [25]. As, in the present study, we did observe changes in the vasoconstriction mediated by U46619, but not by PE or in TP receptor expression, the reduced vasoconstriction to U46619 could be associated with changes in the Rho-kinase pathway; thus, we assessed the protein levels of the main components of this pathway. Briefly, Rho-kinase has two isoforms (ROCK1 and ROCK2) that phosphorylate the subunit of MYPT1, leading to the inhibition of the myosin light chain kinase (MLCK) and vasoconstriction [51]. MYPT1 can be phosphorylated at different sites, but most frequently by ROCK at threonine 855 compared with other phosphorylation sites [52]. Indeed, we found that the phosphorylation levels of MYPT1 at threonine 855 were reduced in the HCD group compared with controls. This suggests there may be an impaired contribution of the Rho-kinase pathway in Ca^2+^handling/sensitization in thoracic aortas from the adult female HCD offspring. Furthermore, MYPT1 can contribute to changes in vascular smooth muscle cell (VSMC) phenotypes, as MYPT1 deficiency led to a phenotype switching from a contractile to a synthetic phenotype in VSMCs of cerebral cortical arteries [53]. VSMC phenotype switching has also been implicated in the development of cardiovascular disease, including atherosclerosis [54]; thus, it may be speculated that the reduced vasoconstriction responses in thoracic aortas from the adult female HCD offspring could be explained by MYPT1-mediated VSMC phenotype switching. The thoracic aorta is a major site for the development of atherosclerotic plaque [55], and in apolipoprotein E knockout (ApoE^−/−^) mice, maternal hypercholesterolemia induced by a Western diet prior to pregnancy and throughout gestation and lactation increased atherosclerotic lesions of the aorta in only the adult female offspring and was associated with changes in aortic macrophage polarization and a proinflammatory phenotype [56]. Thus, collectively, it appears that female offspring may be more susceptible to the impact of maternal hypercholesterolemia on future atherosclerosis development.

In contrast, in male, but not female, offspring, we observed that a HCD in pregnancy reduced endothelium-dependent vasodilation to MCh in only the coronary arteries. Endothelium-dependent vasodilation in the coronary arteries is almost completely NO-mediated [57]. NO is synthesized in the endothelium by nitric oxide synthases (NOS) and then diffuses to the VSMCs to induce relaxation [21]. We did not observe any differences in eNOS or iNOS; however, NO can also react with superoxide, a potent ROS, leading to reduced NO bioavailability [58]. Indeed, we observed higher ROS levels in coronary arteries from the adult male offspring exposed to a HCD in pregnancy, which may lead to reduced NO bioavailability and could explain the reduced endothelium-dependent vasodilation in these male offspring. Higher ROS could be due to higher production or reduced antioxidant capacities [59]. The main intracellular ROS sources include the mitochondria (a natural byproduct of electron transport chain activity [60]) and nicotinamide adenine dinucleotide phosphate oxidases (or Nox) [61]. NOS can also produce superoxide instead of NO when dysfunctional due to the lack of essential cofactors or oxidative stress [62]. The reaction of NO and superoxide can lead to the formation of peroxynitrite, which has been associated with cardiovascular pathologies [57,58,63]. However, we did not observe changes in the levels of 3-nitrotyrosine, a common footprint of peroxynitrite production. We speculate that when HCD coronary arteries are stimulated with MCh, the already increased basal ROS levels may increase further, thereby contributing to the observed endothelial dysfunction.

Interestingly, while *in vivo* cardiac function was comparable between the CD and HCD groups in both sexes, the cardiac susceptibility to an I/R insult (modeling the cardiac capacity to recover from a secondary hit) was increased in *only* the adult male HCD offspring compared with controls. As we also showed impaired endothelium-dependent vasodilation in the coronary arteries of these males, it may be speculated that this impacted function of the coronary arteries could have contributed to the reduced cardiac capacity to recover from the I/R insult, but additional studies are warranted to determine this causative association. Interestingly, in a retrospective human study, maternal hypercholesterolemia in pregnancy was associated with the severity of acute myocardial infarction in both male and female adults [64]. Similarly, other studies from our group reported that prenatal hypoxia, another common pregnancy complication, reduced cardiac capacity to recover from ischemia in both male and female adult offspring [33,65]. Thus, the impact of excessive hypercholesterolemia in pregnancy on cardiac performance of the offspring, and how males and females are affected, differs based on the type of adverse stimulus during prenatal life.

In summary, the present study provides physiological and molecular evidence that excessively high levels of cholesterol during pregnancy affect the cardiovascular health of the adult offspring in a vascular bed- and sex-specific manner. The assessment of maternal lipid profile throughout pregnancy could be a beneficial tool to identify offspring who may have an increased susceptibility to secondary hits throughout their life and who may be at higher risk of developing cardiovascular disease later in life.

## Supplementary material

online supplementary material 1.

## Data Availability

The data supporting the findings of this study are available from the corresponding author upon reasonable request.

## Abbreviations

AUC: area under the curve
BSA: bovine serum albumin
CCRC: cumulative concentration–response curve
CD: control diet
Ca^2+^: calcium
DHE: dihydroethidium
GD: gestational day
HBSS: Hank’s Balanced Salt Solution
HCD: high cholesterol diet
I/R: ischemia/reperfusion
KPSS: K^+^ physiological salt solution
MCh: methacholine
MFI: mean fluorescent intensity
MLCK: myosin light chain kinase
MYPT1: myosin phosphatase target subunit 1
NO: nitric oxide
NOS: nitric oxide synthase
PBS: phosphate-buffered saline
PBST: phosphate-buffered saline with 0.1% Tween 20
PE: phenylephrine
PKC/CPI-17: protein kinase C-potentiated myosin phosphatase inhibitor of 17 kDa
PSS: physiological salt solution
ROCK1: Rho-associated coiled-coil kinase 1
ROCK2: Rho-associated coiled-coil kinase 2
ROS: reactive oxygen species
SEM: standard error of the mean
TP: thromboxane prostanoid
TxA_2_: thromboxane A_2_
VSMC: vascular smooth muscle cell
a.u.: arbitrary units
eNOS: endothelial nitric oxide synthase
iNOS: inducible nitric oxide synthase
pMYPT1: myosin phosphatase target subunit 1 phosphorylated at threonine 855
