# Supplementary material for: Excessive hypercholesterolemia in pregnancy impairs the cardiovascular health of the adult female and male offspring
Source: Biosci Rep. 2026 Jan 9;46(1):BSR20253861. doi: 10.1042/BSR20253861 (PMC12863029; doi:10.1042/BSR20253861)
Supplement: online supplementary material 1. [file bsr-46-1-BSR20253861-s001.docx]

**SUPPLEMENTAL MATERIAL**

**Excessive hypercholesterolemia in pregnancy impairs the cardiovascular health of the adult female and male offspring**

Murilo E. Graton^1,2^, Amanda A. de Oliveira^1,2^, Aryan Neupane^2,3^, Raven Kirschenman^1,2^, Anita Quon^1,2^, Floor Spaans^1,2^, Christy-Lynn M. Cooke^1,2^, Sandra T. Davidge^1,2,3,*^

^1^Department of Obstetrics and Gynecology, University of Alberta, Edmonton, AB, Canada

^2^Women and Children's Health Research Institute, University of Alberta, Edmonton, AB, Canada

^3^Department of Physiology, University of Alberta, Edmonton, AB, Canada

*Corresponding author:

Sandra T. Davidge, PhD, FCAHS, FRSC

Distinguished University Professor

University of Alberta, 232 Heritage Medical Research Centre

Edmonton, Alberta, T6G 2S2, Canada

E-mail: [sandra.davidge@ualberta.ca](mailto:sandra.davidge@ualberta.ca)

ORCID: 0000-0002-5559-4905**Supplementary Table 1.** Vasoconstriction responses to a high K^+^ physiological salt solution in mesenteric, carotid and coronary arteries, and thoracic aortas of the adult female and male offspring born from control diet (CD) pregnancies or high cholesterol diet (HCD) pregnancies.

| **Vascular bed** | **CD** | **HCD** | **Unpaired t-test** |
| --- | --- | --- | --- |
| **Female offspring** | | | |
| Mesenteric arteries | 7.6±0.3 (n=11) | 7.8±0.5 (n=8) | p=0.6918 |
| Carotid arteries | 5.7±0.2 (n=11) | 5.4±0.4 (n=8) | p=0.4784 |
| Thoracic aorta | 12.7±0.3 (n=11) | 12.3±0.5 (n=7) | p=0.4610 |
| Coronary arteries | 3.6±0.4 (n=11) | 4.4±0.2 (n=8) | p=0.1549 |
| **Male offspring** | | | |
| Mesenteric arteries | 8.8±0.6 (n=11) | 9.1±0.4 (n=8) | p=0.7361 |
| Carotid arteries | 6.0±0.3 (n=11) | 6.6±0.7 (n=8) | p=0.4331 |
| Thoracic aorta | 12.5±0.3 (n=11) | 13.3±0.9 (n=7) | p=0.3989 |
| Coronary arteries | 5.5±0.2 (n=11) | 4.8±0.3 (n=8) | p=0.1594 |

Data are presented as means±SEM of the maximum vasoconstriction (mN/mm), and were analyzed with an unpaired Student’s t-test (n=7-11/group; one offspring/sex/dam/group).

**Supplementary Table 2.** Vasoconstriction responses to U46619 in mesenteric, carotid and coronary arteries of the adult female and male offspring born from control diet (CD) pregnancies or high cholesterol diet (HCD) pregnancies.

| **Vascular bed** | **CD** | **HCD** | **Unpaired t-test** |
| --- | --- | --- | --- |
| **Female offspring** | | | |
| Mesenteric arteries | 13.1±2.2 (n=8) | 15.3±2.2 (n=7) | p=0.4915 |
| Carotid arteries | 21.0±0.8 (n=11) | 20.7±3.3 (n=5) | p=0.7446 |
| Coronary arteries | 10.4±1.6 (n=11) | 12.8±1.5 (n=8) | p=0.3151 |
| **Male offspring** | | | |
| Mesenteric arteries | 13.6±2.3 (n=11) | 20.7±3.3 (n=8) | p=0.1019 |
| Carotid arteries | 22.3±1.4 (n=11) | 23.1±1.6 (n=4) | p=0.9216 |
| Coronary arteries | 17.1±1.4 (n=11) | 14.1±1.4 (n=8) | p=0.1731 |

Data are presented as means±SEM of the area under the curve (in arbitrary units), and were analyzed with an unpaired Student’s t-test (n=4-11/group; one offspring/sex/dam/group).

**Supplementary Table 3.** Vasoconstriction responses to phenylephrine in mesenteric and carotid arteries, and thoracic aortas, of the adult female and male offspring born from control diet (CD) pregnancies or high cholesterol diet (HCD) pregnancies.

| **Vascular bed** | **CD** | **HCD** | **Unpaired t-test** |
| --- | --- | --- | --- |
| **Female offspring** | | | |
| Mesenteric arteries | 11.9±0.8 (n=11) | 13.3±1.4 (n=8) | p=0.4044 |
| Carotid arteries | 10.2±1.1 (n=9) | 9.4±1.1 (n=7) | p=0.6278 |
| Thoracic aorta | 32.6±1.9 (n=11) | 27.2±2.8 (n=8) | p=0.1244 |
| **Male offspring** | | | |
| Mesenteric arteries | 15.4±1.0 (n=10) | 16.0±1.0 (n=8) | p=0.7387 |
| Carotid arteries | 12.0±1.2 (n=8) | 13.8±1.2 (n=7) | p=0.3366 |
| Thoracic aorta | 41.2±1.8 (n=11) | 43.2±2.7 (n=7) | p=0.5386 |

Data are presented as means±SEM of the area under the curve (in arbitrary units), and were analyzed with an unpaired Student’s t-test (n=7-11/group; one offspring/sex/dam/group).**Supplementary Table 4.** Vasorelaxation responses to methacholine in thoracic aortas, mesenteric and carotid arteries, of the adult female and male offspring born from control diet (CD) pregnancies or high cholesterol diet (HCD) pregnancies.

| **Vascular bed** | **CD** | **HCD** | **Unpaired t-test** |
| --- | --- | --- | --- |
| **Female offspring** | | | |
| Mesenteric arteries | 396.2±22.0 (n=8) | 413.4±22.0 (n=7) | p=0.5923 |
| Carotid arteries | 285.0±24.0 (n=9) | 326.9±6.5 (n=7) | p=0.1259 |
| Thoracic aortas | 246.4±15.2 (n=11) | 286.2±17.1 (n=8) | p=0.1030 |
| **Male offspring** | | | |
| Mesenteric arteries | 306.3±18.8 (n=8) | 348.5±14.0 (n=4) | p=0.1750 |
| Carotid arteries | 233.1±26.2 (n=9) | 277.1±25.1 (n=8) | p=0.2467 |
| Thoracic aortas | 205.9±18.0 (n=11) | 206.0±18.0 (n=8) | p=0.9960 |

Data are presented as means±SEM of the area under the curve (in arbitrary units), and were analyzed with an unpaired Student’s t-test (n=4-11/group; one offspring/sex/dam/group).

**Supplementary Table 5.** Cardiac left ventricular morphology, systolic function, diastolic function, and myocardial performance index, assessed by transthoracic echocardiography, of female adult offspring born from control diet (CD) pregnancies or high cholesterol diet (HCD) pregnancies.

| **Outcome** | **CD** | **HCD** | **Unpaired t-test** |
| --- | --- | --- | --- |
| **Female offspring** | | | |
| **Left ventricle morphology** | | | |
| LVID; d (mm) | 6.2±0.2 (n=10) | 6.3±0.2 (n=5) | p=0.8355 |
| LVID; s (mm) | 3.5±0.1 (n=10) | 3.6±0.20 (n=5) | p=0.6253 |
| LVAW; d (mm) | 1.8±0.1 (n=10) | 2.0±0.1 (n=5) | p=0.3839 |
| LVAW; s (mm) | 3.0±0.1 (n=10) | 3.3±0.2 (n=5) | p=0.0935 |
| LVPW; d (mm) | 1.9±0.1 (n=10) | 1.9±0.1 (n=5) | p=0.8344 |
| LVPW; s (mm) | 2.5±0.1 (n=9) | 2.5±0.1 (n=5) | p=0.9961 |
| LV mass (mg) | 815.0±47.7 (n=10) | 896.5±54.5 (n=5) | p=0.3167 |
| **Systolic function** | | | |
| HR (bpm) | 320.3±12.6 (n=10) | 316.3±18.4 (n=5) | p=0.8587 |
| SV (μL) | 151.1±11.0 (n=10) | 150.4±9.2 (n=5) | p=0.9691 |
| EF (%) | 74.2±0.8 (n=10) | 72.7±2.1 (n=5) | p=0.4496 |
| FS (%) | 44.1±0.7 (n=10) | 42.9±1.8 (n=5) | p=0.4998 |
| CO (mL/min/kg) | 47.7±2.8 (n=10) | 47.8±4.7 (n=5) | p=0.9835 |
| LV vol; d (μL) | 203.7±15.3 (n=10) | 207.3±15.1 (n=5) | p=0.8355 |
| LV vol; s (μL) | 52.6±4.5 (n=10) | 56.9±8.0 (n=5) | p=0.6234 |
| **Diastolic function** | | | |
| MV E (mm/s) | 847.7±48.2 (n=10) | 859.9±39.6 (n=5) | p=0.8726 |
| MV A (mm/s) | 610.6±25.7 (n=10) | 646.4±37.3 (n=5) | p=0.4423 |
| MV E/A | 1.3±0.1 (n=10) | 1.3±0.1 (n=5) | p=0.7208 |
| MV AET (ms) | 68.7±4.5 (n=8) | 69.8±2.6 (n=5) | p=0.8561 |
| MV A’ (mm/s) | -50.9±3.8 (n=10) | -55.8±5.4 (n=5) | p=0.4736 |
| MV E’ (mm/s) | -61.0±3.2 (n=10) | -62.5±5.4 (n=5) | p=0.8006 |
| MV IVCT (ms) | 17.9±0.7 (n=9) | 18.2±1.1 (n=5) | p=0.8368 |
| MV IVRT (ms) | 23.5±1.7 (n=10) | 24.7±1.9 (n=5) | p=0.6761 |
| MV A’/E’ | 0.8±0.1 (n=10) | 0.9±0.1 (n=5) | p=0.6019 |
| MV E’/A’ | 1.2±0.1 (n=10) | 1.2±0.2 (n=5) | p=0.8026 |
| MV E/E’ | -14.1±0.9 (n=10) | -13.9±0.7 (n=5) | p=0.8830 |
| **Myocardial performance index** | | | |
| Tei index | 0.6±0.1 (n=8) | 0.6±0.1 (n=5) | p=0.7588 |

Data are presented as means±SEM, and were analyzed with an unpaired Student’s t-test (n=5-10/group; one offspring/sex/dam/group). A: Atrial wave velocity, A’: Peak atrial wave velocity, AET: Aortic ejection time, bpm: Beats per minute, CO: Cardiac output, E: Early wave velocity, E’: Peak early wave velocity, EF: Ejection fraction, FS: Fractional shortening, HR: Heart rate, IVCT: Isovolumetric contraction time, IVRT: Isovolumetric relaxation time, LV mass: Calculated left ventricular mass, LV vol; d: Left ventricular volume during diastole, LV vol; s: Left ventricular volume during systole, LVAW; d: Left ventricular anterior wall during diastole, LVAW; s: Left ventricular anterior wall during systole, LVID; d: Left ventricular internal diameter during diastole, LVID; s: Left ventricular internal diameter during systole, LVPW; d: Left ventricular posterior wall during diastole, LVPW; s: Left ventricular posterior wall during systole, ms: Milliseconds, MV: Mitral valve, SV: Stroke volume.

**Supplementary Table 6.** Cardiac left ventricle morphology, systolic function, diastolic function, and myocardial performance index, assessed by transthoracic echocardiography, of male adult offspring born from control diet (CD) pregnancies or high cholesterol diet (HCD) pregnancies.

| **Outcome** | **CD** | **HCD** | **Unpaired t-test** |
| --- | --- | --- | --- |
| **Male offspring** | | | |
| **Left ventricle morphology** | | | |
| LVID; d (mm) | 7.7±0.2 (n=8) | 7.7±0.4 (n=8) | p=0.8525 |
| LVID; s (mm) | 4.2±0.2 (n=8) | 4.4±0.1 (n=8) | p=0.3513 |
| LVAW; d (mm) | 2.1±0.1 (n=8) | 2.1±0.1 (n=8) | p=0.7497 |
| LVAW; s (mm) | 3.6±0.1 (n=8) | 3.5±0.1 (n=8) | p=0.8092 |
| LVPW; d (mm) | 2.1±0.2 (n=8) | 2.4±0.1 (n=8) | p=0.2864 |
| LVPW; s (mm) | 3.1±0.3 (n=8) | 3.4±0.2 (n=8) | p=0.5524 |
| LV mass (mg) | 1340.0±97.3 (n=8) | 1528.0±70.1 (n=8) | p=0.1396 |
| **Systolic function** | | | |
| HR (bpm) | 305.0±9.8 (n=8) | 272.2±16.5 (n=8) | p=0.1106 |
| SV (μL) | 238.2±12.0 (n=8) | 232.9±1 (n=8) | p=0.7578 |
| EF (%) | 74.5±1.7 (n=8) | 71.3±0.9 (n=8) | p=0.1179 |
| FS (%) | 42.5±0.6 (n=8) | 42.1±0.8 (n=8) | p=0.1130 |
| CO (mL/min/kg) | 72.7±4.8 (n=8) | 64.1±6.2 (n=8) | p=0.2926 |
| LV vol; d (μL) | 322.3±21.7 (n=8) | 325.6±41.2 (n=8) | p=0.9020 |
| LV vol; s (μL) | 84.1±10.3 (n=8) | 92.7±3.4 (n=8) | p=0.4433 |
| **Diastolic function** | | | |
| MV E (mm/s) | 786.8±44.1 (n=8) | 783.2±53.1 (n=6) | p=0.9598 |
| MV A (mm/s) | 592.0±29.8 (n=8) | 549.4±33.1 (n=7) | p=0.3512 |
| MV E/A | 1.3±0.1 (n=8) | 1.6±0.1 (n=7) | p=0.1840 |
| MV AET (ms) | 65.8±5.2 (n=7) | 75.0±4.8 (n=6) | p=0.2342 |
| MV A’ (mm/s) | -57.6±2.2 (n=8) | -48.2±4.1 (n=7) | p=0.0583 |
| MV E’ (mm/s) | -60.5±3.4 (n=8) | -56.9±5.2 (n=7) | p=0.5647 |
| MV IVCT (ms) | 21.0±3.0 (n=8) | 20.5±1.3 (n=7) | p=0.9090 |
| MV IVRT (ms) | 24.0±2.1 (n=8) | 25.3±1.3 (n=6) | p=0.6516 |
| MV A’/E’ | 0.9±0.1 (n=8) | 0.8±0.1 (n=7) | p=0.2640 |
| MV E’/A’ | 1.0±0.1 (n=8) | 1.1±0.1 (n=7) | p=0.2716 |
| MV E/E’ | -13.6±1.7 (n=8) | -17.0±3.1 (n=7) | p=0.3430 |
| **Myocardial performance index** | | | |
| Tei index | 0.7±0.1 (n=7) | 0.6±0.1 (n=5) | p=0.5560 |

Data are presented as means±SEM, and were analyzed with an unpaired Student’s t-test (n=5-8/group; one offspring/sex/dam/group). A: Atrial wave velocity, A’: Peak atrial wave velocity, AET: Aortic ejection time, bpm: Beats per minute, CO: Cardiac output, E: Early wave velocity, E’: Peak early wave velocity, EF: Ejection fraction, FS: Fractional shortening, HR: Heart rate, IVCT: Isovolumetric contraction time, IVRT: Isovolumetric relaxation time, LV mass: Calculated left ventricular mass, LV vol; d: Left ventricular volume during diastole, LV vol; s: Left ventricular volume during systole, LVAW; d: Left ventricular anterior wall during diastole, LVAW; s: Left ventricular anterior wall during systole, LVID; d: Left ventricular internal diameter during diastole, LVID; s: Left ventricular internal diameter during systole, LVPW; d: Left ventricular posterior wall during diastole, LVPW; s: Left ventricular posterior wall during systole, ms: Milliseconds, MV: Mitral valve, SV: Stroke volume.

**Supplementary Figure 1.** Representative blots (A) and densitometry of myosin phosphatase target subunit 1 (MYPT1) phosphorylated at Thr^855^ (pMYPT1, B; normalized to MYPT1) and MYPT1 (C; normalized to total protein) in thoracic aortas from the adult male offspring born from control diet (CD; black symbols) pregnancies or high cholesterol diet (HCD; orange symbols) pregnancies. Data are presented as means±SEM and were analyzed with an unpaired Student’s t-test (n=5-6/group; one offspring/dam/group).

**Supplementary Figure 2.** Representative images (*i*) and quantitative analysis (*ii*) of dihydroethidium (DHE) in coronary artery sections from the adult female offspring born from control diet (CD; black symbols) pregnancies or high cholesterol diet (HCD; orange symbols) pregnancies. Data are presented as means±SEM, and were analyzed with an unpaired Student’s t-test (n=5-6/group; one offspring/dam/group). a.u. = arbitrary units, MFI = mean fluorescent intensity.

**Supplementary Figure 3.** Full nitrocellulose membranes for assessment of thromboxane prostanoid receptor protein expression in thoracic aortas from the adult female offspring born from control diet (CD) pregnancies or high cholesterol diet (HCD) pregnancies. Western blotting images for target protein (35-75 kDa within dashed lines, top) and respective total protein stain (bottom). L: Protein ladder, n=6/group; one offspring/dam/group.

**Supplementary Figure 4.** Full nitrocellulose membranes for assessment of Rho-associated coiled-coil kinase 1 protein expression in thoracic aortas from the adult female offspring born from control diet (CD) pregnancies or high cholesterol diet (HCD) pregnancies. Western blotting images for target protein (~161 kDa within dashed lines, top) and respective total protein stain (bottom). L: Protein ladder, n=6/group; one offspring/dam/group.

**Supplementary Figure 5.** Full nitrocellulose membranes for assessment of Rho-associated coiled-coil kinase 2 protein expression in thoracic aortas from the adult female offspring born from control diet (CD) pregnancies or high cholesterol diet (HCD) pregnancies. Western blotting images for target protein (~161 kDa within dashed lines, top) and respective total protein stain (bottom). L: Protein ladder, n=6/group; one offspring/dam/group.

**Supplementary Figure 6.** Full nitrocellulose membranes for assessment of phosphorylated myosin phosphatase target subunit (MYPT1) 1 at threonine 855 (pMYPT1) and total MYPT1 protein expression in thoracic aortas from the adult female offspring born from control diet (CD) pregnancies or high cholesterol diet (HCD) pregnancies. Western blotting images for target protein (~130 kDa within dashed lines, top for pMYPT1 and middle for MYPT1) and respective total protein stain (bottom). L: Protein ladder, n=6/group; one offspring/dam/group.

**Supplementary Figure 7.** Full nitrocellulose membranes for assessment of phosphorylated myosin phosphatase target subunit (MYPT1) 1 at threonine 855 (pMYPT1) and total MYPT1 protein expression in thoracic aortas from the adult male offspring born from control diet (CD) pregnancies or high cholesterol diet (HCD) pregnancies. Western blotting images for target protein (~130 kDa within dashed lines, top for pMYPT1 and middle for MYPT1) and respective total protein stain (bottom). L: Protein ladder, n=6/group; one offspring/dam/group.
